# Rethinking the Gut-Bone Axis: Diet, the Gut Ecosystem, and Skeletal Health

**DOI:** 10.1007/s11914-026-00990-1

**Published:** 2026-10-26

**Authors:** Laura R. McCabe

**Affiliations:** Department of Physiology, Michigan State University, Biomedical Physical Science Building, 567 Willson Road, East Lansing, MI 48824 USA

**Keywords:** Osteoporosis, Gut-bone axis, Gut microbiome, Gut ecosystem, Diet and bone health, Intestinal barrier Integrity

## Abstract

**Purpose of Review:**

The gut–bone field has expanded beyond its initial focus on the gut microbiota to include microbial metabolism, epithelial barrier integrity, immune and endocrine signaling, and host–microbial interactions. This review uses a functional gut ecosystem framework to integrate these mechanisms and examine how diet and other exposures influence skeletal health.

**Recent Findings:**

No consistent microbial signature of osteoporosis has emerged across populations or experimental models. Instead, recent studies indicate that skeletal responses are associated with functional outputs of the gut ecosystem, including nutrient bioavailability, microbial co-metabolic products, barrier integrity, and host sensing. Gut-derived signals may act directly on bone or be further processed by the host. Diverse interventions, including whole foods, probiotics, postbiotics, and targeted therapies, can converge on these functions.

**Summary:**

Bone responds to the integrated biological signals generated by the gut ecosystem rather than microbial composition alone. Defining these functions and individual responder phenotypes may provide new approaches for preserving skeletal health.

## Introduction

Osteoporosis is a multifactorial skeletal disease characterized by reduced bone mass, deterioration of bone microarchitecture, and increased susceptibility to fracture. Aging, estrogen deficiency, genetics, physical activity, and nutrition are well-established determinants of skeletal health. However, over the past decade, the gastrointestinal tract has emerged as an additional important regulator of bone remodeling, extending the conventional view of the intestine from a simple site of nutrient absorption to a dynamic source of metabolic, immune, and endocrine signals [1, 2].

Early work on the gut-bone axis focused on the gut microbiota and microbial composition. Germ-free models, antibiotic-mediated microbiota depletion, fecal microbiota transplantation, probiotics, and dietary interventions demonstrated that changes within the intestinal microbial environment can modify bone mass, remodeling, and quality [3–6]. These studies established a causal role for the gut microbiota in skeletal regulation and stimulated a rapidly expanding field of investigation. The scope of the field is substantial, with a recent systematic review including 248 studies spanning microbiome-bone mechanisms, clinical associations, and therapeutic interventions [1]. Despite this extensive body of work, the field has struggled to identify a consistent microbial signature of osteoporosis.

Taxa associated with low bone mass differ across populations, animal models, diets, medications, and analytical platforms [1]. Similar skeletal phenotypes have been associated with markedly different microbial communities, while differences in microbial composition may not closely track the severity of the skeletal phenotype. For example, Rettedal et al. [7] found significant differences in microbial composition between women with normal bone density and those with osteopenia or osteoporosis but did not detect significant differences between the osteopenic and osteoporotic groups despite significant differences in BMD and T-scores. These observations suggest that microbial composition alone is unlikely to provide a complete understanding of skeletal regulation. Rather, the biological signals that influence the skeleton arise from the integrated function of the intestinal ecosystem, where the person and their gut microbes continuously interact to generate metabolic, immune, and endocrine signals that influence skeletal physiology through multiple pathways.

This review presents a functional and integrated gut ecosystem framework in which diet and other exposures interact with both the individual and their gut microbes to generate biological signals. The term gut ecosystem is used to describe this integrated physiological network, which includes dietary exposures, resident microorganisms, host-microbial co-metabolism, epithelial barrier function, immune regulation, and endocrine signaling. The review examines how these biological signals generated by the ecosystem influence skeletal health and how they may be modified to support bone health.

## The Gut Ecosystem as a Functional Interface

The intestine forms a key interface between the external environment of the intestinal lumen and the host internal environment. Food and dietary substrates entering the lumen are exposed to digestive enzymes, bile acids, transport systems, epithelial cells, immune cells, enteroendocrine signals, and a metabolically diverse microbial community. Some compounds are absorbed directly, but many others undergo host-microbial co-metabolism, whereby microbial and host enzymatic processes sequentially transform dietary substrates before they enter the circulation. The biological exposure experienced by bone therefore reflects not only what is consumed, but how the gut ecosystem processes what is consumed. Thus, the intestine is not simply a site of nutrient absorption but an active physiological system that processes dietary and environmental exposures into signals that can influence peripheral tissues, including bone.

This perspective helps explain why similar diets may produce different physiological responses. Emerging evidence indicates that host genetics, intestinal transit time, bile acid composition, and baseline ecosystem composition all contribute to variability in microbial metabolism and responsiveness to dietary or probiotic interventions [8–11]. Genetic disruption of bile acid conjugation, for example, simultaneously alters nutrient absorption, microbial community development, the intestinal metabolome, and systemic physiology, demonstrating that host and microbial metabolism operate as one interconnected system [12].

**Fig. 1:**
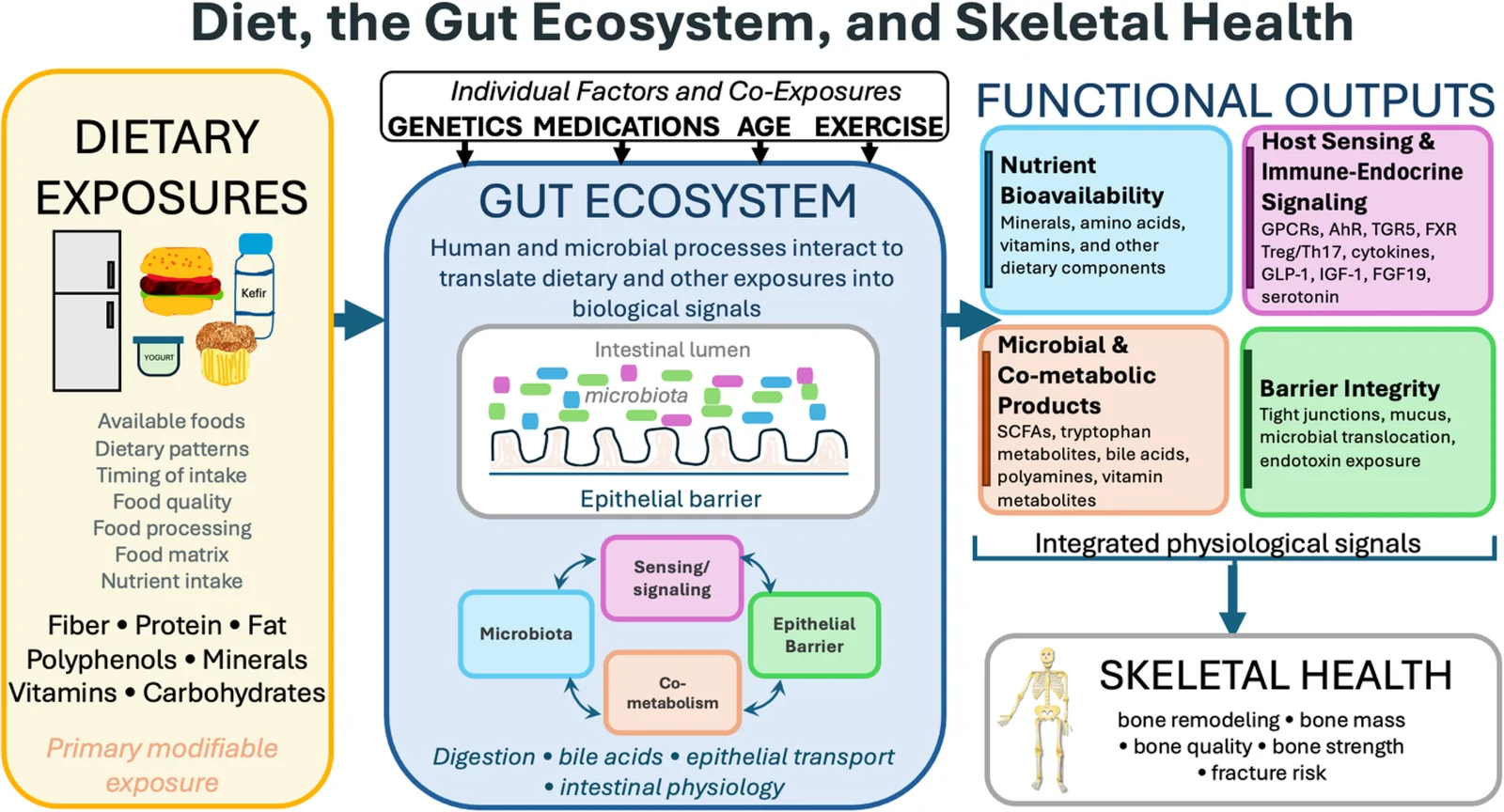
Conceptual framework illustrating how dietary and other exposures are translated by the interacting human and microbial processes of the gut ecosystem into functional biological outputs that influence skeletal health. Abbreviations: GPCR, G protein-coupled receptor; AhR, aryl hydrocarbon receptor; TGR5, Takeda G protein-coupled receptor 5; FXR, farnesoid X receptor; Treg, regulatory T cells; Th17, T helper 17 cells; GIP, glucose-dependent insulinotropic polypeptide; GLP-1, glucagon-like peptide-1; IGF-1, insulin-like growth factor 1; FGF19, fibroblast growth factor 19

Figure 1 summarizes the conceptual framework presented in this review. Diet and other exposures are integrated by the gut ecosystem to generate four major functional outputs: nutrient bioavailability, microbial co-metabolic products, barrier integrity, and host sensing with immune-endocrine signaling. These functional outputs, their principal skeletal links, and representative supporting evidence are summarized in Table 1. Although presented as distinct functional outputs, these interconnected functions operate as an integrated physiological network that ultimately regulates bone remodeling, bone mass, bone strength, and fracture risk.

**Table 1:** Major functional outputs of the gut ecosystem that influence skeletal health

Functional output	Representative processes or signals	Principal skeletal links	Illustrative evidence	Conceptual implication
Nutrient bioavailability	Food-matrix breakdown; digestion; bile acid–mediated micelle formation; epithelial transport; host metabolism; microbial transformation of nutrients	Determines delivery and utilization of calcium, vitamin D, amino acids, minerals, and other substrates required for remodeling and mineralization	Neugebauer et al. 2022; Richards et al. 2025	Biological exposure is not determined solely by dietary intake; nutrient availability reflects integrated host and microbial processing.
Microbial and co-metabolic products	SCFAs; secondary bile acids; indoles; polyamines; vitamins; valerate; imidazole propionate; extracellular vesicles	Direct regulation of osteoclasts and osteoblasts; Treg–WNT10B signaling; marrow adipogenesis; host receptor-mediated signaling; communication beyond soluble metabolites	Lucas et al. 2018; Tyagi et al. 2018; Lin et al. 2023; Park et al. 2024; Lyu et al. 2024; Liu et al. 2021	Function is more informative than taxonomy: distinct communities may generate similar outputs, whereas similar communities may differ metabolically.
Barrier integrity	Tight junctions; mucus; epithelial turnover; antimicrobial defenses; control of microbial and LPS translocation	Limits systemic exposure to microbial products; restrains inflammatory and osteoclastogenic signaling; preserves skeletal homeostasis	Li et al. 2016; Collins et al. 2017; Schepper et al. 2019, 2020; Chargo et al. 2023; Yu et al. 2024; Tian et al. 2025; Shieh et al. 2026	The barrier is both an output of microbial metabolism and a determinant of which microbial signals reach the host.
Host sensing and immune–endocrine integration	GPCRs; AhR; TGR5; FXR; LBP–CD14–TLR4; cytokines; Treg/Th17 balance; IGF-1; GIP; GLP-1; GLP-2; serotonin; FGF19	Translates gut-derived signals into changes in osteoclastogenesis, osteoblast activity, postprandial remodeling, growth, and systemic inflammation	Yan et al. 2016; Li et al. 2016; Tyagi et al. 2018, 2021; Bouvard and Mabilleau 2024; Chen et al. 2024; Zheng et al. 2025; Tume et al. 2025	Host sensing determines how gut-derived signals are interpreted and translated into skeletal responses.

Categories are separated for conceptual clarity but interact continuously in vivo. References are illustrative rather than exhaustive; broad field syntheses include Ticinesi et al. (2025) and Ohlsson et al. (2026). Abbreviations: *AhR* aryl hydrocarbon receptor, *FXR* farnesoid X receptor, *GPCR* G protein-coupled receptor, *IGF-1* insulin-like growth factor 1, *LPS* lipopolysaccharide, *SCFA* short-chain fatty acid, *TGR5* Takeda G protein-coupled receptor 5, *Treg* regulatory T cell

## Diet and Other Individual Exposures Shape Gut Ecosystem Function

Diet is the principal modifiable exposure shaping the gut ecosystem because it determines the substrates entering the intestinal tract. Dietary exposure begins even before ingestion, because an individual’s food environment – including the availability, accessibility, timing, and source of foods - influences what is consumed and therefore determines the substrates ultimately presented to the gut ecosystem. Dietary intake, however, should not be equated with biological exposure. Nutrient bioavailability depends on food matrix, digestion, microbial transformation, epithelial transport, host metabolism, and tissue utilization. Consequently, the nutritional value of a food is determined not only by what it contains but also by how efficiently the gut ecosystem converts those nutrients into biologically available substrates [13].

Traditional nutritional approaches to bone emphasize calcium, vitamin D, protein, phosphorus, magnesium, and total energy intake. These factors remain fundamental. However, whole foods also deliver fiber, fermentable carbohydrates, polyphenols, lipids, vitamins, minerals, and other bioactive compounds within a physical matrix that influences digestion, absorption, and microbial transformation. Dietary composition can favor the growth of specific microbial populations, but importantly, it also affects what those microorganisms produce and how they function. Fermentable fibers support short-chain fatty acid production; dietary protein supplies substrates for indoles, polyamines, imidazole propionate, and other amino-acid-derived metabolites; dietary fat alters bile acid pools and microbial ecology; and polyphenols are converted into metabolites that can differ substantially from their parent compounds. Thus, diet influences which organisms are present, the biological products they generate, and how those products are sensed by the individual [1, 2].

Diet simultaneously modifies aspects of the intestinal environment such as mucus production, epithelial turnover, luminal pH, intestinal transit, barrier integrity, and immune tone. A single dietary intervention can consequently affect several pathways at once. Whole foods illustrate this particularly well. Prunes, for example, provide fiber, sorbitol, polyphenols, minerals, and vitamins within an intact food matrix. Their skeletal effects are therefore unlikely to derive from a single constituent and may reflect coordinated changes in nutrient handling, microbial metabolism, inflammation, and intestinal physiology [14].

Dietary exposure also has a temporal dimension. When food is consumed can affect circadian rhythms, microbial activity, and skeletal physiology. In a recent study, long-term time-restricted feeding during the normal rest phase caused gut dysbiosis and bone loss in male mice and was associated with reduced fecal propionate and increased gut microbiota-dependent Th17 signaling. Fecal microbial transplantation supported a causal contribution of the altered microbiota to the immune and skeletal response [15]. These findings suggest that the timing of intake may be another component of dietary exposure that influences the gut ecosystem. Human evidence directly linking meal timing with gut-mediated skeletal outcomes remains limited.

Another important concept is that diet does not act in isolation. Aging, medications, physical activity, obesity, chronic disease, and genetics also shape gut ecosystem function. Antibiotics and many non-antimicrobial drugs can alter microbial diversity and metabolic output, whereas glucocorticoids can disrupt both intestinal and skeletal physiology [16–19]. Mechanical loading associated with physical activity likewise interacts with the gut ecosystem to influence skeletal adaptation [20, 21]. In mice, differences in skeletal responsiveness to mechanical loading were associated with distinct gut microbial communities, and the high-responder phenotype was transferable by fecal microbiota transplantation. Microbial arginine metabolism was identified as one mechanism contributing to the enhanced osteocyte response to loading [21]. Together, these findings illustrate that diet and other individual exposures interact to shape gut ecosystem function and influence how environmental stimuli are translated into skeletal responses.

## Functional Pathways Linking the Gut Ecosystem to Bone

### Nutrient Bioavailability: More Than Absorption

The best-established intestinal contribution to skeletal health is the digestion and absorption of nutrients required for bone maintenance. As discussed above, nutrient bioavailability reflects not simply dietary intake but the integrated processes of food matrix breakdown, digestion, microbial transformation, epithelial transport, and host metabolism. Consequently, the amount of a nutrient consumed does not necessarily equal the amount ultimately delivered to the skeleton.

Bile acids provide a useful example. They facilitate dietary fat digestion by promoting micelle formation and are critical for the absorption of fat-soluble vitamins (including vitamins D and K) while simultaneously functioning as signaling molecules and ecological determinants of the intestinal environment. Neugebauer and colleagues demonstrated that disruption of bile acid conjugation impaired nutrient handling, altered microbial community development, and reshaped systemic metabolism [12]. This illustrates that nutrient bioavailability reflects the combined effects of digestion, epithelial transport, microbial activity, and enterohepatic metabolism rather than simply dietary content.

### Microbial Metabolism Translates Diet into Biological Signals

Host-microbial co-metabolism – the coordinated processing of dietary and other compounds by the individual and their gut microorganisms - is a defining function of the gut ecosystem. Through fermentation and other metabolic processes, the intestine generates short-chain fatty acids, secondary bile acids, indoles, polyamines, vitamins, and numerous other bioactive products capable of acting locally or entering the circulation. Short-chain fatty acids provide one of the best-established examples. They can directly inhibit osteoclast activity and stimulate bone formation through regulatory T-cell-dependent WNT10B signaling [22, 23]. Butyrate-producing organisms have also preserved bone in experimental models, supporting function rather than taxonomic identity as the more relevant target [24]. Short-chain fatty acids, however, represent only one group of microbial products.

The diversity of microbial effects becomes even more apparent when considering amino-acid metabolism. Microbial production of tryptophan-derived indoles supports epithelial integrity and can influence osteoblast and osteoclast biology, whereas the histidine-derived imidazole propionate suppresses osteoblast differentiation and promotes marrow adipogenesis through p38γ-AMPK signaling [25]. Integrated human multi-omics and experimental studies have further linked reduced valeric acid production with osteoporosis through a Bacteroides vulgatus-valerate pathway [26]. These findings show that microbial metabolites are not inherently beneficial; skeletal effects depend on the type, amount, and balance of products generated. Taxonomically different microbial communities could therefore produce similar skeletal outcomes when they generate similar functional outputs, whereas apparently similar communities may differ substantially in their effects because of differences in metabolite profiles. Finally, gut-bone communication may extend beyond soluble metabolites. Extracellular vesicles released by gut bacteria are an emerging route of communication and experimental work suggests that these vesicles can reach bone and provide another way for the microbiota to influence osteoblast and osteoclast activity [27].

### Barrier Integrity Maintains the Host-Microbial Interface

The intestinal barrier is a dynamic interface between the outside environment of the intestinal lumen and the inside of the body. It is composed of epithelial cells, tight junctions, mucus, antimicrobial products, and resident immune cells. It permits nutrient absorption while limiting excessive passage of microorganisms and microbial products into host tissues. When barrier integrity is disrupted, exposure to endotoxin and other microbial signals increases, which can amplify systemic inflammation and osteoclastogenic pathways [28].

Evidence from estrogen-deficiency models indicates that barrier dysfunction is an important upstream contributor to skeletal deterioration. Estrogen deficiency increases intestinal permeability, alters epithelial and immune signaling, and promotes microbiota-dependent inflammatory responses that contribute to bone loss [6, 29, 30]. Recent longitudinal human data provide evidence that similar changes occur across the menopausal transition. In 964 women followed before, during, and after menopause [31], intestinal fatty acid-binding protein 2 (FABP2), a marker of epithelial injury, began increasing approximately 2.5 years before the final menstrual period, followed by an increase in soluble CD14 (sCD14), a marker of microbial product-related immune activation. Both remained elevated after menopause and increases in FABP2 over time were associated with increases in sCD14 in the same women. These findings provide important human evidence that intestinal barrier disruption associated with estrogen deficiency in experimental models may also occur across the menopausal transition.

Interventions that preserve or restore epithelial integrity can reduce inflammatory responses and improve skeletal outcomes across multiple experimental models. Barrier-enhancing polymers have prevented bone loss in models involving post-antibiotic dysbiosis and glucocorticoid exposure [16, 17]. Colon-targeted postbiotic nanoparticles also restored claudin-1 and ZO-1 expression, reduced circulating lipopolysaccharide and inflammatory cytokines, and improved bone mass and strength [32]. Systemically administered irisin similarly improved intestinal barrier function and attenuated ovariectomy-induced bone loss in mice [30].

Glucocorticoid models provide particularly strong evidence for this relationship. Glucocorticoid-induced trabecular bone loss is microbiota dependent and associated with increased intestinal permeability, whereas depletion of the gut microbiota, restoration of barrier function, or treatment with Lactobacillus reuteri 6475 prevented bone loss [17]. In a clinically relevant oral glucocorticoid model, L. reuteri 6475 prevented the glucocorticoid-induced increase in circulating lipopolysaccharide (LPS) and preserved trabecular bone, whereas a multi-strain probiotic produced less skeletal protection [33]. Together, these studies identify intestinal barrier integrity as a modifiable contributor to glucocorticoid-induced skeletal deterioration.

Microbial metabolism and barrier integrity are also closely linked. Microbial metabolites can strengthen or impair epithelial integrity. For example, engineered tryptophan-producing bacteria restored tight-junction proteins, reduced inflammation, and mitigated bone loss in colitis and aging models [34]. Barrier integrity can therefore be both an output of microbial metabolism and a determinant of which microbial signals reach the body.

### Host Sensing Integrates Microbial Signals into Skeletal Physiology

Microbial metabolism determines which biological signals are produced, but how each individual senses these signals ultimately determines whether and how they are translated into a skeletal response. Microbial products may act directly on skeletal cells or engage receptors expressed in epithelial, immune, hepatic, endocrine, and skeletal tissues. Gut-derived signals may also be metabolized, modified, or relayed by other organs before ultimately influencing the skeleton. Thus, the skeletal response depends not only on the signal itself but also on how it is processed and sensed by the individual. Host sensing is the critical translation layer between gut ecosystem function and skeletal physiology.

G protein-coupled receptors (GPCRs), the aryl hydrocarbon receptor (AhR), bile acid receptors such as Takeda G protein-coupled receptor 5 (TGR5) and farnesoid X receptor (FXR), and pattern-recognition receptors including the lipopolysaccharide-binding protein (LBP)-cluster of differentiation 14 (CD14)-Toll-like receptor 4 (TLR4) complex are among the principal systems that translate intestinal chemistry into changes in bone remodeling [35, 36].

Bile acid metabolites provide a clear example of this principle. The HDCA-TGR5 pathway links microbial metabolism, host receptor activation, and skeletal phenotype. In a mouse study, aging was associated with reduced Parabacteroides goldsteinii, lower concentrations of hyodeoxycholic acid (HDCA), and bone loss. Fecal microbiota transplantation from young donors increased HDCA availability and preserved bone mass by suppressing osteoclastogenesis. These protective effects were markedly attenuated in TGR5-deficient mice, directly linking microbial metabolism with host sensing [37].

Host sensing through the AhR pathway provides a second example. Microbial indole metabolites improved intestinal barrier integrity and prevented ovariectomy-induced bone loss through activation of epithelial AhR signaling. Loss of intestinal AhR largely abolished these protective effects, demonstrating that production of a metabolite alone is not sufficient; host sensing is also required to translate microbial metabolism into a skeletal response [38].

Host sensing also extends beyond microbial metabolites to structural microbial components such as lipopolysaccharide (LPS). Barrier dysfunction increases systemic exposure to luminal LPS, where recognition through the LBP-CD14-TLR4 complex activates inflammatory pathways that enhance osteoclast signaling and contribute to bone loss [28]. Interventions that preserve barrier integrity or reduce circulating LPS levels attenuate these responses and prevent bone loss in experimental models [16–18]. Importantly, structurally distinct LPS molecules can elicit different host responses [35], suggesting that the biological properties of microbial signals, not simply their abundance, also influence skeletal physiology.

Host sensing converges with immune and endocrine regulation. Short-chain fatty acids (SCFAs) promote regulatory T-cell expansion, whereas sex steroid deficiency enhances microbiota-dependent inflammatory cytokines and bone loss [5, 6, 22]. The gut ecosystem also regulates circulating insulin-like growth factor 1 (IGF-1) [4]. In addition, enteroendocrine hormones such as glucose-dependent insulinotropic polypeptide (GIP), glucagon-like peptide 1 (GLP-1), and glucagon-like peptide 2 (GLP-2) translate nutrient ingestion into rapid systemic signals that influence postprandial bone remodeling [39]. Together, these findings suggest that the intestine functions as an integrated sensory organ, interpreting dietary and microbial information and converting it into coordinated immune, endocrine, and metabolic signals that regulate skeletal physiology.

## Modulating Gut Ecosystem Function to Support Skeletal Health

Recognition that bone responds to gut ecosystem function has stimulated the development of diverse therapeutic strategies aimed at modifying the intestinal environment. These approaches include probiotics, prebiotics, postbiotics, whole foods, engineered microorganisms, and receptor-targeted therapies. Although they differ in what they deliver, many converge on the same functional outputs: microbial metabolism, barrier integrity, host sensing, and immune-endocrine regulation. Representative intervention strategies, targeted ecosystem functions, skeletal outcomes, and key limitations are summarized in Table 2.

**Table 2:** Representative strategies for restoring gut ecosystem function to support skeletal health

Strategy	Representative example(s)	Evidence base / model	Gut ecosystem function(s) targeted	Reported skeletal outcome	Key qualification(s) and representative references
Probiotics	Lactobacillus and Bifidobacterium formulations; L. reuteri 6475	OVX rodent meta-analysis; glucocorticoid and post-antibiotic models; human meta-analysis and RCTs	Microbial composition and metabolism; barrier integrity; immune signaling	Generally favorable preclinical effects on BMD and trabecular structure; human findings mixed	Effects are strain-, host-, and context-dependent. Bose and Sharan 2024; Schepper et al. 2019, 2020; Chargo et al. 2023; Wang et al. 2024; Resciniti et al. 2026.
Prebiotics and fermentable fibers	FOS/GOS and other fermentable substrates	Predominantly preclinical studies	Substrate availability; microbial metabolism; SCFA production; barrier and immune regulation	Improved mineral absorption and/or bone outcomes in selected models	Mechanisms and effective formulations remain incompletely defined; effects depend on the resident ecosystem.
Whole foods	Prunes; blackcurrants	OVX, glucocorticoid, and disuse models; randomized human trials	Nutrient bioavailability; microbial metabolism; barrier function; inflammatory signaling; host sensing	Prunes preserve bone in several preclinical models; 50 g/day preserved total hip BMD in postmenopausal women; blackcurrant pilot trial attenuated whole-body bone loss	Complex food matrices engage multiple pathways; responder heterogeneity is important. Smith et al. 2022; De Souza et al. 2022; Chargo et al. 2023; Simpson et al. 2024; Nosal et al. 2024; Hanzawa et al. 2025; George et al. 2022; Ormsbee et al. 2026.
Postbiotics and defined metabolites	SCFAs; HDCA; other defined microbial products	Mechanistic animal and cellular studies	Direct delivery of functional products; host receptor activation	Reduced osteoclastogenesis and/or improved bone mass in experimental systems	Dose, stability, tissue exposure, and off-target effects require definition. Lucas et al. 2018; Zheng et al. 2025.
Engineered microorganisms	Tryptophan-producing bacteria	Colitis and aging models	Restoration of defined metabolic activity; barrier repair; immune modulation	Restored tight-junction proteins, reduced inflammation, and mitigated bone loss	Remains preclinical; safety, durability, containment, and regulatory pathways require study. Tian et al. 2025.
Barrier-targeted delivery	Barrier-enhancing polymers; colon-targeted postbiotic nanoparticles	Post-antibiotic dysbiosis, glucocorticoid, OVX, and barrier-injury models	Epithelial barrier restoration; reduced LPS translocation; localized delivery	Reduced inflammation and preserved or improved bone outcomes	Supports barrier restoration as a therapeutic mechanism, but platforms remain experimental. Schepper et al. 2019, 2020; Yu et al. 2024.
Targeted signaling and advanced delivery	HDCA–TGR5 targeting; bacterial extracellular vesicles; targeted oral delivery systems	Preclinical mechanistic and delivery studies	Host sensing; cell-specific or tissue-directed delivery; host–microbial communication	Suppressed osteoclastogenesis, preserved bone mass/strength, or improved marrow-cell function	Highly promising but predominantly preclinical; causal pathway specificity and translation remain to be established. Liu et al. 2021; Qu et al. 2025; Zheng et al. 2025.

Representative rather than exhaustive. *OVX* ovariectomy, *BMD* bone mineral density, *FOS* fructooligosaccharides, *GOS* galactooligosaccharides, *SCFA* short-chain fatty acid, *HDCA* hyodeoxycholic acid, *LPS* lipopolysaccharide. In the text, TRAP5b refers to tartrate-resistant acid phosphatase 5b

Initial efforts focused on modulation of the gut microbiota through probiotics and prebiotics. Preclinical evidence for probiotics is strong. A meta-analysis of 22 ovariectomized rodent studies demonstrated significant improvements in bone mineral density and trabecular bone volume despite substantial heterogeneity in strains and study design [40]. Clinical syntheses also suggest potential benefit. A meta-analysis of 12 randomized trials involving 1,183 postmenopausal women reported improved lumbar spine and hip bone mineral density, particularly in women with osteopenia [41]. However, individual clinical trials remain inconsistent. In a 12-month randomized trial of 114 early postmenopausal women, a lactobacilli formulation did not attenuate bone loss and had no significant effects on bone turnover or inflammatory markers [42]. Prebiotics and fermentable fibers complement these approaches by modifying the substrates available to resident microorganisms, thereby altering microbial metabolism rather than directly introducing new organisms.

Whole-food interventions represent a broader ecosystem-based strategy because they simultaneously influence multiple gut ecosystem functions. Rather than targeting a single pathway, foods such as prunes and blackcurrants may influence nutrient bioavailability, microbial metabolism, barrier integrity, inflammatory signaling, and host sensing through their complex food matrices. Prunes have one of the broadest experimental evidence bases, with skeletal protection reported across multiple models, including estrogen deficiency, glucocorticoid exposure, and disuse [14, 18, 43]. Dietary prune prevented glucocorticoid-induced trabecular bone loss and produced dose-dependent increases in bone volume in mice [18]. Prune extract also preserved trabecular bone structure and osteoblast-related gene expression in a disuse rat model, although gut-mediated mechanisms were not examined [43]. Other food-derived interventions provide additional evidence that complex dietary bioactives can influence bone through the gut ecosystem. Korean red ginseng extract prevented vertebral trabecular bone loss in an oral glucocorticoid model while altering gut microbial composition, with more modest effects on barrier and immune outcomes [44]. Similarly, six months of blackcurrant supplementation attenuated whole-body bone loss in peri- and early postmenopausal women while altering selected gut microbial taxa and producing trends toward lower IL-1β and RANKL responses. Changes in several bacterial genera were also correlated with changes in whole-body bone mineral density in exploratory analyses, supporting the concept that polyphenol-rich whole foods influence bone through coordinated effects on the gut ecosystem rather than through a single bioactive constituent [45].

Human studies further suggest that responses to ecosystem-targeted dietary interventions are heterogeneous. In postmenopausal women, consumption of 50 g/day of prunes for 12 months preserved total hip bone mineral density relative to controls and maintained fracture-risk estimates [46]. Secondary analyses suggested that women classified as responders exhibited greater abundance of gut Lachnospiraceae, providing an association between gut microbial features and variability in skeletal outcomes [47]. In contrast, short-term studies in older men demonstrated favorable changes in selected bone turnover markers, whereas a one-year randomized trial found no improvement in total or lumbar spine bone mineral density beyond calcium and vitamin D supplementation, although exploratory differences were observed in osteoprotegerin and TRAP5b [48, 49]. Together, these findings suggest that understanding responder phenotypes may be as important as identifying effective interventions.

The field is now moving beyond broad modulation of microbial communities toward therapies that directly restore specific gut ecosystem functions. Postbiotics and defined microbial metabolites seek to deliver functional microbial products without requiring live organisms, while engineered microorganisms are being developed to restore specific metabolic activities, such as tryptophan production [34]. Likewise, emerging therapeutic concepts – including targeting the HDCA–TGR5 pathway, bacterial extracellular vesicles, and colon-targeted hydrogel or nanoparticle systems—illustrate a shift toward precise manipulation of host–microbial communication rather than modification of community composition [27, 32, 37, 50]. Recent reviews similarly emphasize second-generation probiotics, synbiotics, engineered fibers, targeted postbiotics, and species-specific microbiome therapies designed to restore defined ecosystem functions rather than simply reshape microbial composition [51]. The therapeutic goal is therefore shifting from creating a universal “healthy microbiome” to restoring the gut ecosystem functions that support skeletal homeostasis.

## Future Directions and Perspectives

A key translational challenge remains the absence of a reproducible microbial taxonomic signature of skeletal health. Microbial composition varies with geography, diet, age, medications, disease, host genetics, and analytical methodology. Future studies should therefore move beyond sequencing alone by integrating metabolomics, transcriptomics, stable isotope tracing, barrier measurements, immune phenotyping, and skeletal outcomes. Functional biomarkers are likely to prove more reproducible and informative. Identifying host-microbial co-metabolic pathways may be particularly valuable because these pathways integrate microbial function with individual physiology and could define ecosystem states associated with skeletal health. Guild-based analysis is one approach that may help address this problem. Rather than testing individual taxa independently, guild methods group microorganisms that show consistent co-abundance patterns and may contribute to shared ecological functions, reducing the dimensionality and sparsity of microbiome data [52, 53]. These approaches are promising, but guild definition and validation remain active methodological questions.

Establishing causality is also a challenge because most interventions simultaneously influence multiple ecosystem functions. An important next step will be to determine whether gut-derived signals act directly on skeletal tissues or are first processed by other organs and tissues, including the liver, kidney, brain, muscle, and immune system before reaching or influencing bone. Considering these interactions could reveal pathways that are missed when the gut-bone axis is viewed as direct communication between the intestine and skeleton. Future studies should also look beyond bone mass alone. Emerging evidence suggests that gut ecosystem function may also influence muscle mass, muscle function, falls, and ultimately fracture risk. Including these outcomes in dietary and microbiome intervention studies may provide a more complete picture of musculoskeletal health and clinical benefit [51].

Individual variability should also be viewed not simply as a challenge, but as an opportunity to uncover mechanisms. Individuals consuming the same diet or probiotic can generate different microbial metabolites because of differences in microbial metabolic capacity, genetics, intestinal physiology, transit time, medications, and immune status. Understanding why individuals respond differently may reveal the ecosystem functions responsible for skeletal benefit. Precision nutrition and personalized microbiome interventions should therefore focus on identifying responder phenotypes rather than assuming that everyone will respond in the same way [21, 47].

The gut–bone field has progressed from an emphasis on nutrient absorption, to microbial taxonomy, and now toward ecosystem function. Dietary exposures, microbial metabolism, epithelial integrity, immune regulation, endocrine signaling, and host sensing form a coordinated physiological network that determines the biological signals ultimately reaching the skeleton. Thinking about these processes together helps explain why different microbial communities can produce similar skeletal outcomes and why very different interventions can affect bone through common pathways. Bone, therefore, responds not simply to nutrients or microorganisms themselves, but to the biological signals generated as diet and other exposures are processed by the gut ecosystem. The biologically relevant unit is therefore not the microbiota in isolation, but the functional gut ecosystem itself. Understanding how this ecosystem functions, why it differs among individuals, and how it can be modified may provide new opportunities to preserve skeletal and musculoskeletal health.

## Data Availability

No datasets were generated or analysed during the current study.
